# Protein modification regulated autophagy in *Bombyx mori* and *Drosophila melanogaster*

**DOI:** 10.3389/fphys.2023.1281555

**Published:** 2023-11-09

**Authors:** Wenmei Wu, Luobin Lin, Yuntao Zhao, Huaqin Li, Rongxin Zhang

**Affiliations:** ^1^ School of Life Sciences and Biopharmaceuticals, Guangdong Pharmaceutical University, Guangzhou, Guangdong, China; ^2^ Guangzhou Xinhua University, Guangzhou, Guangdong, China

**Keywords:** protein modification, autophagy, *Bombyx mori*, *Drosophila melanogaster*, insects

## Abstract

Post-translational modifications refer to the chemical alterations of proteins following their biosynthesis, leading to changes in protein properties. These modifications, which encompass acetylation, phosphorylation, methylation, SUMOylation, ubiquitination, and others, are pivotal in a myriad of cellular functions. Macroautophagy, also known as autophagy, is a major degradation of intracellular components to cope with stress conditions and strictly regulated by nutrient depletion, insulin signaling, and energy production in mammals. Intriguingly, in insects, 20-hydroxyecdysone signaling predominantly stimulates the expression of most autophagy-related genes while concurrently inhibiting mTOR activity, thereby initiating autophagy. In this review, we will outline post-translational modification-regulated autophagy in insects, including *Bombyx mori* and *Drosophila melanogaster*, in brief. A more profound understanding of the biological significance of post-translational modifications in autophagy machinery not only unveils novel opportunities for autophagy intervention strategies but also illuminates their potential roles in development, cell differentiation, and the process of learning and memory processes in both insects and mammals.

## Introduction

Autophagy is a universally conserved catabolic mechanism present in all eukaryotic cells. This process facilitates the degradation of intracellular entities, such as damaged organelles and proteins, by transporting them to lysosomes or vacuoles. The primary objective of autophagy is to recycle cytoplasmic materials during environmental stress conditions (Maiuri et al., 2007; Klionsky et al., 2021). In addition to its role in cellular renewal, metabolism, physiology, inflammation, and homeostasis, autophagy also significantly influences the regulation of the immune response and contributes to our understanding of the pathogenesis of human disorders (Mizushima and Levine, 2010; Rabinowitz and White, 2010; Levine et al., 2011). Based on the delivery pathways to the lysosome, autophagy is categorized into three primary types: macroautophagy, microautophagy, and chaperone-mediated autophagy (Klionsky and Emr, 2000). Furthermore, autophagy is strictly regulated by conditions such as nutrient starvation, energy signaling, cellular stresses, and virus infection in mammals and yeast (Ravanan et al., 2017; Dikic and Elazar, 2018; Hua et al., 2019; Mao et al., 2019). However, in insects, such as *B. mori* (*Bombyx mori*) and *D. melanogaster* (*Drosophila melanogaster*), a unique regulator of autophagy during metamorphosis phases is 20E (20-hydroxyecdysone), a steroid hormone synthesized from dietary cholesterol. 20E either induces the expression of *Atgs* (autophagy-related genes) or inhibits mTORC1 activity (McPhee and Baehrecke, 2009; Li et al., 2016; Guo et al., 2018; Li et al., 2019; Dai et al., 2020). Macroautophagy, commonly referred to autophagy, involves the biogenesis of autophagosomes, which is orchestrated by a series of *Atgs* (Tian et al., 2013; Klionsky et al., 2021). These *Atgs* were originally identified in *Saccharomyces cerevisiae* and are highly conserved across species, from insects to mammals (Chang and Neufeld, 2009; Araki et al., 2013; Tian et al., 2013; Tettamanti and Casartelli, 2019). The maturation of autophagosomes involves their fusion with the lysosome, leading to the formation of the autolysosome, which elicits the digestion of cellular components (Klionsky et al., 2021). Recently, several PTMs (post-translational modifications) have been reported in addition to acetylation/deacetylation, phosphorylation/dephosphorylation, ubiquitination/deubiquitination, and other modifications, all of which play a role in regulating autophagic activities in yeast, insects, and mammals (Popelka and Klionsky, 2015; Xie et al., 2015; Wang and Wang, 2019). However, it is worth noting that the accuracy of PTMs in regulating autophagy occurrence can vary among different species (Yi et al., 2012; Banreti et al., 2013).

The silkworm, *B. mori*, belonging to the Lepidoptera order, holds significant commercial value in the silk industry and serves as a representative model insect for agricultural and genetic research (Li et al., 2016; Li et al., 2019; Dai et al., 2020). Beyond its economic importance, *B. mori* provides a wide range of cellular and molecular tools, making it indispensable for the study of autophagy during metamorphosis and its role in post-embryonic development (Xie et al., 2016; Guo et al., 2018; Montali et al., 2019). On the other hand, the fruit fly, *D. melanogaster*, a dipteran insect, has become one of the favored models in cell and developmental biology due to its short reproductive cycle, robust genetics, cost-effective maintenance, and ease of manipulation (Rubin, 1988). In *D. melanogaster*, tissues such as the midgut, intersegmental muscles, and fat body undergo programmed cell death, with autophagy playing a pivotal role in their degradation and regeneration (McPhee and Baehrecke, 2009; Guo et al., 2019; Shi and Tong, 2022). This organism also provides a genetically tractable system for exploring the role of autophagy in human disease models, including Parkinson, Alzheimer, and Pick’s disease (Arias, 2008; Chang and Neufeld, 2009; Chanu and Sarkar, 2020; Denton et al., 2020). Furthermore, it has been reported that the protective role of spermidine, an endogenous polyamine, against age-induced memory impairment has been elucidated using the *D. melanogaster* model. This work has revealed that age-related memory decline can be alleviated through spermidine treatment and genetic enhancements to polyamine levels. The underlying mechanism appears to be linked to autophagy-a cellular cleanup process. These findings suggest that a diet rich in polyamine-containing foods may offer a potential therapeutic approach to counteract age-related dementia. However, it is crucial to conduct further research to validate these findings beyond the fruit fly model (Sigrist et al., 2014).

In this review, we will delve into the PTMs that play a role in regulating autophagy in insects, with a particular emphasis on *B. mori* and *D. melanogaster*. Additionally, we will present novel perspectives on the intricate interplay between these sophisticated PTM mechanisms and the processes of learning and memory in insects. Understanding how these PTMs regulated autophagy will not only illuminate the fundamental mechanisms underlying autophagy in insects but also provide unique insights into the processes of development and cell differentiation, both in insects and mammals.

## PTMs mediated autophagy in *B. mori* and *D. melanogaster*

### Acetylation and deacetylation

**FIGURE 1:**
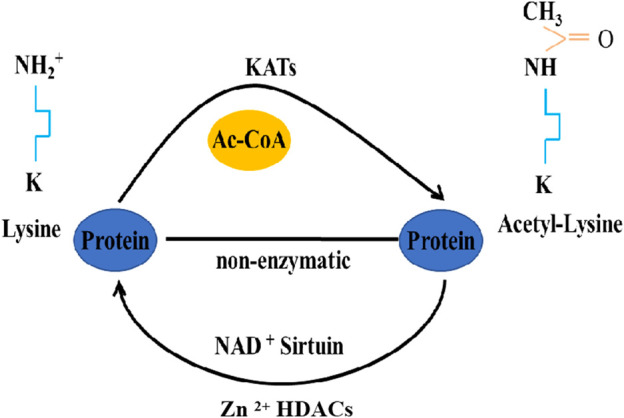
Acetylation/deacetylation pathways in eukaryotes.

Histone acetyltransferases (HATs) and histone deacetylases (HDACs) are enzymes responsible for catalyzing acetylation and deacetylation of lysine residues on histone and non-histone proteins, respectively (Ropero and Esteller, 2007; Chen et al., 2015). Specifically, HATs transfer an acetyl group from acetyl-coenzyme A to the ɛ-amino group of an internal lysine residue, while HDACs reverse these reactions (Figure 1). In mammals, there are 13 identified HATs, categorized into three types based on their structure and function: p300 (E1A binding protein p300), GCN5 (General control non-derepressible 5), and the MYST19 family (Sapountzi and Côté, 2011; Drazic et al., 2016; Narita et al., 2019). HDACs are further classified into several classes: class I, IIa, IIb, and IV enzymes are zinc-dependent histone deacetylases, while class III enzymes are NAD^+^-dependent sirtuins (Yang and Seto, 2008; Haberland et al., 2009). Crucially, both HATs and HDACs play pivotal roles in regulating various cellular processes, including replication, DNA damage repair, cell cycle, apoptosis, angiogenesis, and autophagy (Narita et al., 2019). Current research indicates that HATs and HDACs are integral to autophagy regulation, influencing it at multiple stages (Fullgrabe et al., 2013; Huang et al., 2015). This review delves into the regulation of autophagy in insects through acetylation and deacetylation modifications.

The silk moth *B. mori* exhibits pronounced autophagy during its molting and metamorphic stages, particularly in the silk gland and fat body (Tian et al., 2013; Xie et al., 2016; Li et al., 2020). The initiation and completion of autophagosome formation involve the Atg5–Atg12–Atg16L1 ubiquitin-like systems and the protein light chain 3 (LC3)/Atg8-PE (phosphatidylethanolamine) (Ichimura et al., 2000; Kaiser et al., 2012; Schaaf et al., 2016). The LC3/Atg8-PE conjugation is primarily catalyzed in sequence by Atg4, Atg7, Atg3, and Atg8 (Schaaf et al., 2016). Wu et al. (2021a) emphasized that the autophagy regulation through acetylation/deacetylation is activated by Atg proteins. This evidence preliminarily suggests that acetylation of *Atg* plays a role in autophagy regulation in the silkworm. However, these studies do not fully elucidate the regulation of autophagy. Are there additional genes involved? What other specific regulatory effects exist? Answers to these questions can provide a deeper understanding of the role acetylation plays in autophagy. In the HATs class, P300 mediates acetylation that sequesters components of the Atg8-PE ubiquitin-like system in the nucleus, consequently hindering the process of autophagy (Narita et al., 2019). In contrast, HDAC1/Rpd3 (histone deacetylase 1/reduced potassium dependency 3), a class I enzyme that includes zinc-dependent histone deacetylases, mediates deacetylation (Haberland et al., 2009; Narita et al., 2019). This deacetylation process leads to the nucleo-cytoplasmic translocation of the Atg8-PE ubiquitin-like system components, including Atg4, Atg7, Atg3, and Atg8, thereby promoting autophagy (Wu et al., 2021a). Within the intricate tapestry of cellular regulation, the mechanism through which HDAC1/Rpd3 overexpression enhances autophagy remains consistent between insects and mammals. However, in a striking divergence, yeast intensifies starvation-induced autophagy upon the deletion of deacetylase Rpd3, highlighting the intricate nuances of autophagic processes across diverse species (Yi et al., 2012; Huang et al., 2015). Despite the widespread recognition of p300 and HDAC1 in regulating acetylation, there is a notable scarcity of studies investigating their specific roles in autophagy post-acetylation. Consequently, in-depth research in this area is imperative. Another study has revealed that the acetylation of Atg8, a *B. mori* counterpart of yeast Atg8, is believed to impede the initiation of autophagy during periods of starvation. Specifically, following BmNPV infection, the K13 site of Atg8 undergoes acetylation. Studies utilizing acetylation-mimic K13Q or deacetylation-mimic K13R mutants have demonstrated that this acetylation at K13 inhibits Atg8-PE formation, particularly after EBSS treatment, consequently disrupting the initiation of autophagy (Xue et al., 2019). Furthermore, this acetylation diminishes Atg8’s interaction with Atg7, potentially affecting Atg8-PE conjugation. In essence, the silk moth *B. mori* undergoes significant autophagy during specific life stages, with the process intricately regulated by various proteins and enzymes. The acetylation and deacetylation of these proteins are pivotal in the initiation and progression of autophagy. While some mechanisms are conserved across species, others, such as the acetylation of Atg8, have unique implications in *B. mori*, affecting the onset of autophagy under certain conditions. The Atg8/LC3/GABARAP family members are ubiquitin-like proteins essential to autophagy. They covalently attach to phagophore membranes, playing roles in phagophore elongation and cargo recognition. Importantly, the lipid conjugation of Atg8/LC3/GABARAP is crucial for the formation of autophagosomes–lysosomes (Klionsky et al., 2021). Atg8 serves as a marker for autophagy in insects (Franzetti et al., 2012; Tian et al., 2013; Xie et al., 2016). Additionally, *Drosophila’s* Atg8a protein, analogous to mammalian LC3, orchestrates autophagy by interacting with the transcription factor Sequoia, YL-1 (a nuclear acetyltransferase component), and the deacetylase Sir2 (silent information regulator 2). The latter modulates Atg8a's acetylation status in response to nutrient conditions (Jacomin et al., 2020). It has also been reported that high pH induces the conversion of Atg8-PE in *B. mori* Bme cells rather than amino acid starvation (Gai et al., 2013). *D. melanogaster* species possess two Atg8 homologs, Atg8a and Atg8b (Jipa et al., 2021). While lipidated Atg8a is essential for autophagy in *D. melanogaster*, its non-lipidated form plays a critical role in programmed larval midgut elimination and viability (Bali and Shravage, 2017). In contrast, Atg8b is exclusively found in the male germline and does not impact autophagy. Non-lipidated Atg8b in the male germline is vital for fertility (Jipa et al., 2021). The previously mentioned research has illuminated the significant influence of acetylation on genes that regulate autophagy. However, further exploration is necessary to determine whether individual genes or the collective regulation of gene groups trigger autophagy. These insights have substantial implications, particularly in agricultural production, where site-specific gene modification and multi-target auxiliary control therapies are paramount.

Acetylation and deacetylation not only modulate autophagy-related proteins but also regulate a wider range of proteins involved in autophagy regulation. For example, TFEB (transcription factor EB), which orchestrates the expression of genes crucial for autophagosome formation and lysosome production, plays an integral role in lysosomal biogenesis and activation (Settembre et al., 2011; Martini-Stoica et al., 2016). In *D. melanogaster*, the acetyltransferase GCN5 acetylates TFEB at K274 and K279, disrupting TFEB dimerization and its binding to target gene promoters, thereby inhibiting autophagy and lysosome biogenesis (Wang et al., 2020). These findings collectively emphasize the indispensable role of acetylation and deacetylation in the initial regulation of autophagy. Furthermore, AcCoA (acetyl-coenzyme A), identified as a metabolic suppressor of autophagy (Lu and Thompson, 2012; Kaelin and McKnight, 2013), has been shown to enhance the clearance of autophagic proteins and extend lifespan when knocked down in *D. melanogaster* (Eisenberg et al., 2014). Another study revealed that in wild-type *Drosophila* (w1118), AMI (age-related memory impairment) manifests as diminished learning by middle age. Through intricate *in vivo*, DNA microarray, and behavioral analyses, the study identified mAcon1 (mitochondrial Acon1) as a key factor. While its expression decreased with age, neuronal overexpression extended lifespan and alleviated AMI. This change was associated with notable increases in acetyl-CoA and citrate levels, indicators linked to autophagy. Additionally, there was a distinct correlation between autophagy activity, as measured by shifts in Atg8a-II and p62, and mAcon1 levels. This was further emphasized by an inverse connection with the presynaptic scaffold protein Bruchpilot, ultimately revealing that mAcon1’s modulation of learning and AMI operates through autophagy/mitophagy-mediated neural plasticity (Cho et al., 2021). The enzyme ATP13A2, encoded by the *ATP13A2* gene, orchestrates a protein of the lysosomal transmembrane 5 P-type ATPase family (Ramirez et al., 2006; Tan et al., 2011). This leads to lysosomal abnormalities, mitochondrial dysfunction, and α-synuclein aggregation, thereby impacting autophagy (Grunewald et al., 2012; Usenovic and Krainc, 2012; Tsunemi and Krainc, 2014; Wang et al., 2019). In ATP13A2-deficient cells of *D. melanogaster*, HDAC6 activity was reduced, resulting in increased acetylation and disrupted autophagosome–lysosome fusion (Wang et al., 2019) (Table 1). From another perspective, the relationship between autophagosome function and their respective membrane sites requires further examination. For example, will the ability of autophagosomes to perform autophagy change due to alterations in different organelle membranes and binding sites? This area of research is ripe for exploration. Spermidine, a natural polyamine, has been linked to lifespan extension in flies and worms (Fabrizio et al., 2004; Pucciarelli et al., 2012; Yue et al., 2017). It inhibits HAT activity, leading to the epigenetic deacetylation of histone H3. This, in turn, enhances autophagy, suppresses necrosis, and promotes longevity (Eisenberg et al., 2009). These investigations underscore the intricate role of deacetylation as an essential process for regulating autophagy and, consequently, controlling cellular functions. The CREB (cAMP response element binding protein), which includes the CBP (cyclic adenosine monophosphate response element binding protein) and P300, often referred to as the CBP/P300 complex, emphasizes the significance of acetyltransferases CBP and P300 (Das et al., 2009). They are considered crucial in transcriptional regulation, acting as transcription factors.

**TABLE 1:** Acetylation and deacetylation involved in autophagy.

HAT	Target protein	Acetylation site	HDACs	Impact on autophagy	Species
P300	Atg3	K94, K195	HDAC1	Down; Wu et al. (2021a)	*B. mori*
P300	Atg4	K237, K269	HDAC1	Down; Wu et al. (2021a)	*B. mori*
P300	Atg7	Unknown	HDAC1	Down; Wu et al. (2021a)	*B. mori*
Unknown	Atg8	K13	Unknown	Down; Xue et al. (2019)	*B. mori*
P300	Atg8	K6, K13, K29,	HDAC1	Down; Wu et al. (2021a)	*B. mori*
		K24, K26, K28			
GCN5	TEFB	K274, K279	Unknown	Down; Wang et al. (2020)	*D. melanogaster*
Unknown	Tubulin, cortactin	Unknown	HDAC6	Down; Wang et al. (2019)	*D. melanogaster*
Spermidine	Histone H3	Unknown	Unknown	Down; Wang et al. (2019)	*D. melanogaster*

In summary, protein acetylation and deacetylation are widespread in insects and exert significant influence over fundamental cellular processes. While the aforementioned questions remain unanswered definitively, their impact on autophagy holds profound implications for the entire insect lifecycle.

### Phosphorylation and dephosphorylation

**FIGURE 2:**
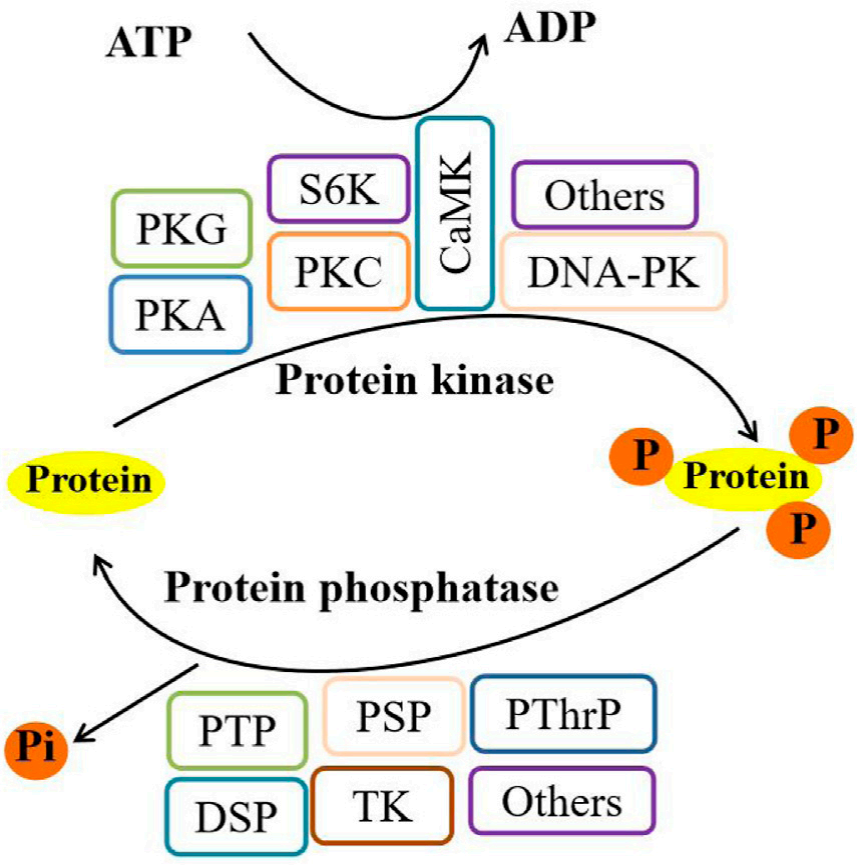
Phosphorylation/dephosphorylation pathways in eukaryotes. Note: PKG, cGMP-protein kinase; PKA, protein kinase A; PKC, protein kinase C; S6K, ribosome S6 kinase; CaMK, calmodulin-dependent protein kinase; DNA-PK, DNA-dependent protein kinase; PTP, protein tyrosine phosphatase; PSP, protein serine phosphatase; PThrP, protein threonine phosphatase; DSP, dual-specificity phosphatase; and TK, tyrosine kinase.

Phosphorylation, a dynamic and reversible process (Figure 2), is catalyzed by protein kinases such as AMPK (AMP-activated protein kinase), PI3K (class III phosphatidylinositol-3 kinase), PKC (protein kinase C), and mTOR (mammalian target of rapamycin) (Qian et al., 2017; Wang et al., 2018; Melick and Jewell, 2020). It involves the attachment of a phosphoryl group to a protein, primarily affecting several amino acids, including Ser (serine), Thr (threonine), Tyr (tyrosine), His (histidine), and Asp (aspartate). Conversely, protein phosphatases are responsible for dephosphorylating proteins (Kim et al., 2011; Denton et al., 2020). Through its modulation of protein function, subcellular localization, and cell signaling, phosphorylation plays a pivotal role in regulating life processes (Singh et al., 2017). In this review, we delve into how phosphorylation and dephosphorylation mediate autophagy in insects, emphasizing the role of autophagy-associated proteins and signaling pathways in autophagy initiation and progression.

In *B*. *mori* and *D*. *melanogaster*, the Atg1 kinase complex, represented by three mRNA isoforms due to alternative splicing, plays a pivotal role in initiating autophagy (Li et al., 2019). This complex responds effectively to reduced insulin signaling, extending lifespan, and highlighting the significance of autophagy in insect longevity. Notably, the phosphorylation of Atg1 by AMPK, especially at Ser269 and Ser270, is essential for autophagy initiation in both species (Li et al., 2022; Zhao et al., 2023). AMPK-mediated phosphorylation of Atg1 is a critical step for inducing autophagy and is evolutionarily conserved among insects. This conservation provides insights into how various organisms regulate ULK1/Atg1 phosphorylation to induce autophagy (Papinski and Kraft, 2016). The autophagy-related protein ATG16L1 is crucial for autophagosome biogenesis (Cadwell et al., 2008). Depletion of *Ptpmeg2* in *D. melanogaster* impairs autophagosome formation and autophagic flux (Chou et al., 2021). Research has observed that Ptpmeg2 co-localizes with ATG16L1 and mediates the dephosphorylation of VTI1B. As a substrate target of Ptpmeg2, this promotes ATG16L1 precursor fusion and autophagosome formation during starvation-induced autophagy (Wang et al., 2016; Chou et al., 2021). Additionally, activated Atg1 can phosphorylate Atg13, Atg17, and itself at positions T226 and S230. Consequently, the phosphorylation state of Atg1 promotes autophagy initiation and facilitates the formation of the Atg1-Atg13-Atg17 complex, which recruits other proteins for phagophore assembly site formation in *D. melanogaster* (Chang and Neufeld, 2009; Davies et al., 2015). By broadly summarizing the relationship between *Atgs* and the functional mechanisms of these proteins, the pivotal role of phosphorylation becomes evident, offering a forward-looking perspective on autophagy regulation.

Beyond modifications to the *Atgs*, other factors have been identified as integral to autophagy regulation. Notably, Tip60 (Tat-interactive protein 60 kDa), a prominent histone acetyltransferase from the MYST family and a member of the nuclear multimeric protein complex, has recently gained attention for its acetylation capabilities. This enzyme is involved in various cellular processes, including the cell cycle, apoptosis, autophagy, and chromatin remodeling, suggesting a novel pathway for autophagy regulation (Ikura et al., 2000; Cheng et al., 2019; Li and Rasmussen, 2020). Starvation-induced AMPK activation leads to the phosphorylation of Tip60 at Ser99, which is essential for autophagy induction in *B. mori*. This mechanism is conserved in lepidopterans and mammals but may not be in other insects (Wu et al., 2020). 20E plays a crucial role in promoting programmed cell death (PCD) by modulating PGK1, a key glycolytic enzyme (Matsui et al., 2012). During metamorphosis, 20E orchestrates a decline in glycolysis and PGK1 expression. Intriguingly, 20E-driven acetylation of PGK1 triggers PCD. Knockdown of phosphorylated PGK1 during the feeding stage results in suppressed glycolysis and smaller pupae. Insulin induces PGK1 deacetylation via HDAC3, while 20E prompts PGK1 acetylation at K386 through ARD1, stimulating PCD (Kang et al., 2023). This intricate interplay between 20E and insulin in regulating PGK1 acetylation underscores a profound connection with autophagy (Kang et al., 2023). Rpd3/HDAC1 may play a role in autophagy regulation, but its pathway requires further exploration. Notably, cholesterol, 27-hydroxycholesterol, and 20E regulate the dephosphorylation of Rpd3/HDAC1 homologs at the serine392, serine421, and serine423 site through MTOR activity and its nucleo-cytoplasmic shuttling, promoting autophagy in *B. mori* and mammals. This suggests potential therapeutic targets for neurodegenerative diseases such as Parkinson’s and Alzheimer’s in humans (Wu et al., 2021b). Additionally, 20E was also found to upregulate the *BmAtg13* gene expression and induce autophagy in *B. mori* (Li et al., 2020). Further studies are required to investigate the potential modification of BmAtg13 sites. Research has shown that impaired PINK1/parkin-mediated mitophagy is a potential molecular basis for mitochondrial abnormalities in Parkinson’s disease (De Rijk et al., 1995; Vives-Bauza et al., 2010; Koyano et al., 2014). In *D. melanogaster*, PINK1-mediated Drp1-S616 phosphorylation induces autophagy, rescuing PINK1 deficiency-associated phenotypes (Han et al., 2020). Collectively, the role of phosphorylation in autophagy regulation is emerging as a promising research direction. Interestingly, the Rag GTPase RagC, a component of the Rag heterodimer, plays a key role in cell growth regulation (Tee et al., 2022). RagC phosphorylation suppresses starvation-induced autophagy in *D. melanogaster*, acting as a positive regulator for mTORC1 activity (Yang et al., 2019). Studies have also revealed that Cdk5, a member of the cyclin-dependent kinases family, is a post-mitotic kinase essential for maintaining neuronal health (Dhavan and Tsai, 2001; Dhariwala and Rajadhyaksha, 2008; Pozo and Bibb, 2016). The direct connection between RagC and Cdk5 in regulating autophagy is not evident, but their shared pathways suggest a potential role in autophagy. Cdk5-induced phosphorylation of Acinus at the serine 437 site, primarily a nuclear protein in *D. melanogaster*, promotes starvation-independent basal autophagy and responses to certain neurodegenerative challenges, confirming a closer link between Cdk5 and autophagy (Nandi et al., 2017) (Table 2). On the other hand, defective brain insulin signaling has been suggested as an early event in Alzheimer’s disease and autophagy (Schnier et al., 1991; Triani et al., 2018), offering a novel perspective on understanding the broad relationship between autophagy and other diseases. In *D. melanogaster* models of autophagy, particularly those expressing the 2N4R-Tau, and in neuroblastoma cells, the downstream effects of the insulin receptor signaling cascade lead to tau hyperphosphorylation at AT8 and PHF1 (PHD finger protein 1) residues (Goncalves et al., 2019). This process is influenced by the interplay of AKT, glycogen synthase kinase-3β, and extracellular regulating kinase, ultimately resulting in aggregation and autophagic defects (Chatterjee et al., 2019). Additionally, the *D. melanogaster* homolog of the human cmyc proto-oncogene has been shown to regulate the phosphorylation status of tauV337M, inducing autophagy (Chanu and Sarkar, 2020). Intriguingly, treatment with β-guanidinopropionic acid, a creatine analog (Reznick and Shulman, 2006; Mukhina et al., 2008), significantly elevated the expression of phospho-T172-AMP-activated protein kinase levels, which, in turn, led to a notable upregulation of the Atg8 protein, inducing autophagy and extending the lifespan of *D. melanogaster* (Yang et al., 2015). JNK or c-Jun N-terminal kinases are a prominent class of protein kinases. These enzymes activate the transcription factor c-Jun, which is a crucial component of the AP-1 transcription factor complex (Zhou and Boutros, 2020). They modify proteins by chemically adding phosphate groups. The JNK pathway is well-known for triggering cytoprotective responses, including autophagy in *D. melanogaster*, showcasing a meticulous regulation of autophagy and offering a fresh perspective (Wu et al., 2009; Brock et al., 2017). JNK opposes insulin signaling, activates the FOXO (Forkhead box protein O), and triggers cytoprotective genes, thereby protecting cells from various stressors (Wang et al., 2005; Kang et al., 2012). Furthermore, JNK signaling in muscular and neural tissues also plays a role in autophagy regulation. Flies with enhanced JNK activity in *Drosophila* exhibit increased stress resilience and longevity. This suggests that JNK signaling can decelerate aging and extend lifespan by promoting autophagy and lysosomal degradation systems, which aid in clearing out damaged protein aggregates (McEwen and Peifer, 2005; Wu et al., 2009; Zeng and Hou, 2015).

**TABLE 2:** Phosphorylation and dephosphorylation involved in autophagy.

Protein kinase	Target protein	Phosphorylation site	Protein phosphate	Impact on autophagy	Species
AMPK	Atg1	S269, S270	Unknown	Up; Zhao et al. (2023)	*B. mori*
AMPK	Tip60	S99	Unknown	Up; Wu et al. (2020)	*B. mori*
mTOR	Rpd3/HDAC1	S392, S421, S423	Unknown	Up; Wu et al. (2021b)	*B. mori*
AMPK	Atg1	S269, S270	Unknown	Up; Zhao et al. (2023)	*D. melanogaster*
mTORC1	TFEB	Unknown	Unknown	Down; Wang et al. (2020)	*D*. *melanogaster*
PINK1	Drp1	S616	Unknown	Up; Han et al. (2020)	*D*. *melanogaster*
Unknown	VTI1B	Unknown	Ptpmeg2,	Up; Chou et al. (2021)	*D*. *melanogaster*
			ATG16L1		
Atg1	Atg1	T226, S230	Unknown	Down; Chang and Neufeld, (2009); Davies et al. (2015)	*D*. *melanogaster*
mTORC1	RagC	S21	Unknown	Down; Yang et al. (2019)	*D*. *melanogaster*
Cdk5	Acinus	S437	Unknown	Up; Nandi et al. (2017)	*D*. *melanogaster*
HDAC3	ARD1	Y194	PGK1	Up; Kang et al. (2023)	*Helicoverpa armigera*

In conclusion, phosphorylation modifications are instrumental in regulating autophagy in both *B. mori* and *D. melanogaster*. These modifications impact various stages of autophagy, including initiation, recognition, execution vesicle formation, autophagosome–lysosome fusion, degradation, and the regulation of other substrates. A deeper comprehension of the interplay between phosphorylation modifications and autophagy has the potential to yield novel insights into research on development and cell differentiation in both humans and insects.

### Ubiquitination and deubiquitination

**FIGURE 3:**
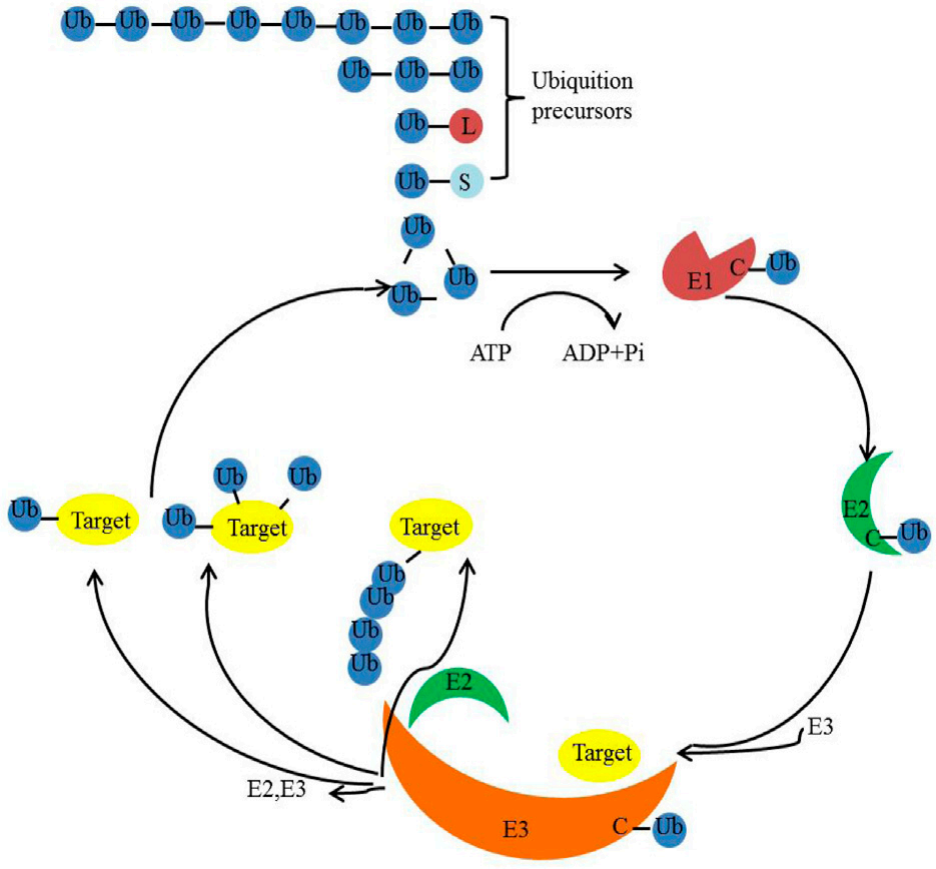
Ubiquitination/deubiquitination pathways in eukaryotes.

Ubiquitination is a post-translational modification present in all eukaryotic organisms. It entails the covalent attachment of ubiquitin, a small protein comprising 76 amino acids, to cellular proteins. Remarkably, this protein is highly conserved across mammals, yeast, and plants (Hershko and Ciechanover, 1998; Varshavsky, 2017). The ubiquitination process is orchestrated by three classes of enzymes: E1 (activation), E2 (conjugation), and E3 (ligation) (Shaid et al., 2013; Gomez-Diaz and Ikeda, 2019) (Figure 3). Ubiquitin itself possesses seven lysine residues and an N-terminal methionine residue, enabling it to bind to another ubiquitin molecule (Ikeda and Dikic, 2008). Beyond controlling physiological and pathological cellular events (Latham and Dent, 2007; Popovic et al., 2014; Hu and Sun, 2016), ubiquitination and deubiquitination also play pivotal roles in governing essential cellular processes. These processes encompass gene transcription, cell migration and differentiation, autophagy, DNA repair, apoptosis, virus budding, and receptor endocytosis (Amit et al., 2004; Latham and Dent, 2007; Hu and Sun, 2016; Schwertman et al., 2016; Grumati and Dikic, 2018; Islam et al., 2018; Rape, 2018; Oh et al., 2020).

p62/SQSTM1 (p62/Sequestosome-1) serves as a key adapter that facilitates the degradation ubiquitinated proteins via the autophagic process. Atg8 homologs are essential for p62-mediated autophagic degradation (Islam et al., 2018). In the lepidopteran insect *B. mori*, p62 bodies consist of PB1 (Phox and Bem1p) and UBA (ubiquitin associated) domains, which are essential for binding and forming p62 bodies. Interestingly, the AIM motif of p62 has the capability to self-associate independently of the PB1 or UBA domain. As previously mentioned, Atg8, which plays an indispensable role in regulating autophagy, is found universally in all eukaryotes (Li and Zhang, 2019; Wang et al., 2021b). In *B. mori*, Atg8’s structure includes an ubiquitin-fold domain at its C-terminus and two additional helices at the N-terminus. This structure is conserved from yeast to mammals. Notably, Atg8 plays a pivotal role in the Atg8-PE ubiquitin-like conjugation system, essential for autophagosome formation in eukaryotes (Hu et al., 2010). PINK1 is a kinase imported into mitochondria. It activates the E3 ubiquitin ligase known as Parkin, which in turn facilitates the selective autophagy of damaged mitochondria (Eiyama and Okamoto, 2015; Lazarou et al., 2015; Nguyen et al., 2016). In *D. melanogaster*, PINK1’s phosphorylation of ubiquitin at serine 65 initiates Parkin’s E3 ubiquitin ligase activity, recruiting Parkin to mitochondria and inducing selective autophagy (Kane et al., 2014). p62 serves as a prototype autophagy adapter protein and facilitated the transports ubiquitinated cargo for subsequent autophagic degradation (Nezis et al., 2008; Johansen and Lamark, 2011). In *D. melanogaster*, enhancing p62 levels during midlife promotes mitochondrial fission, mediates mitophagy, and enhances both mitochondrial function and the lifespan of older flies (Aparicio et al., 2019). VDAC1 (voltage-dependent anion channel protein 1), anchored to the outer mitochondrial membrane, is vital for regulating mitophagy and apoptosis in Parkinson’s disease (Bayrhuber et al., 2008; Geisler et al., 2010; Shoshan-Barmatz et al., 2020). In *D. melanogaster*, VDAC1 exists in two different forms: polyubiquitination and monoubiquitination. In its polyubiquitination form, VDAC1 impedes Parkin-mediated mitophagy. However, in its monoubiquitination form at the K274R site, defective VDAC1 induces apoptosis by augmenting mitochondrial calcium uptake through the mitochondrial calcium uniporter channel (Ham et al., 2020). The USP7 (ubiquitin-specific protease 7), a deubiquitinating enzyme, plays a role in repairing DNA damage and various cancers and organism development (Zlatanou et al., 2016; Su et al., 2018; Pozhidaeva and Bezsonova, 2019; Zhao et al., 2021). Knockdown of USP7 in *D. melanogaster* leads to increased ubiquitination of proteins and monoubiquitin. This indicates that the USP7 is necessary to maintain *D. melanogaster*’s normal lifespan by regulating both autophagy and ubiquitin signaling pathways (Cui et al., 2020). Deubiquitinating enzymes, which remove ubiquitin from proteins, are essential regulators of ubiquitin-dependent processes (Clague et al., 2012). As mentioned earlier, the FOXO in JNK signaling was also reported to promote damaged protein clearance through the autophagy/lysosome system, enhancing muscle function and lifespan (Wang et al., 2005; Kang et al., 2012). This study suggested that muscle-based FOXO/4E-BP signaling reduces feeding and insulin release, subsequently delaying protein aggregate accumulation in various tissues with age (Demontis and Perrimon, 2010). Interestingly, minocycline, when administered to aging *Drosophila*, emerges as a potent agent for improving proteostasis, a crucial component of cellular health. This enhancement is intricately linked to the upregulation of autophagy genes, with the autophagy/lysosomal pathway in *Drosophila* muscles acting as the primary mediators of this protective effect through the FOXO-Hsp70 axis (Lim and Hyun, 2022). Furthermore, UBQLN (ubiquilin) plays a pivotal role in cellular proteostasis due to its role in the ubiquitin proteasome system and autophagy. Mutations in UBQLN2 have been linked to amyotrophic lateral sclerosis and its variant with frontotemporal lobar dementia (ALS/FTLD) (Gilpin et al., 2015). *Drosophila* possesses a dUbqn (UBQLN homolog) akin to UBQLN1 and UBQLN2. This study has revealed that depletion of dUbqn leads to the accumulation of polyubiquitinated proteins accumulation and morphological tissue anomalies, with specific neural defects correlating with hindered locomotion, learning, and memory. This illuminates the impact of proteostasis disruption in neurodegenerative diseases and offers a promising *Drosophila* model for ALS and FTLD therapeutic exploration (Jantrapirom et al., 2018).

In essence, the ubiquitin-mediated autophagy process is a critical cellular mechanism that ensures the timely degradation and recycling of cellular components. Its dysregulation can have profound implications for cellular health and function. By delving deeper into the intricacies of this pathway and its involvement in disease pathology, researchers can lay the foundation for innovative therapeutic approaches applicable to a variety of diseases in both humans and insects. The potential for targeted treatments that modulate this pathway offers hope for improved patient outcomes and an improved quality of life for those affected by diseases associated with autophagy dysregulation.

### Others

Protein lipidation is a crucial post-translational modification that attaches lipids to proteins, thereby influencing their membrane localization and function (Chen et al., 2018; Czuba et al., 2018). This modification holds particular significance in the context of autophagy, a cellular degradation process. In the *B. mori*, the lipidation of the BmAtg8 is essential for the degradation of p62 bodies, a process mediated by autophagy (Ji et al., 2017). The membrane localization of these proteins is governed by their covalent modifications with distinct lipids. In addition, studies have shown that the lipidation of BmAtg8, specifically BmAtg8-PE, is essential for the autophagic degradation of p62 bodies in *Bombyx* cells. Moreover, the lipidation of BmAtg8 does not influence the interaction between p62 and BmAtg8 (Ji et al., 2017). This leads to interactions with other proteins, consequently affecting their conformation, stability, membrane association, and localization (Resh, 2013; Maeda et al., 2019; Ragland et al., 2020). The broader implications of protein lipidation extend beyond autophagy, as it can reshape protein, enhance their stability, and alter their interactions with various cellular components. This can have profound effects on cellular processes and has been linked to various diseases. For instance, abnormalities in protein lipidation can contribute to neurological disorders, metabolic diseases, cancers, and a range of other conditions (Chen et al., 2016).

Hypusination is a unique post-translational modification wherein the amino acid lysine undergoes transformation into the atypical amino acid, hypusine, in eukaryotic organisms (Cooper et al., 1983). The significance of hypusination is further accentuated by its occurrence in a specific protein, the eIF5A (eukaryotic initiation factor 5A) (Schnier et al., 1991). The role of eIF5A is pivotal in protein synthesis, and the presence of hypusine is believed to be essential for its function in *D. melanogaster* (Patel et al., 2009).

Histone methylation significantly influences the transcriptional status of genes. This modification is site-specific and can be reversed by histone lysine methyltransferases and lysine demethylases (Latham and Dent, 2007). Methylation of histone 3 lysine 4, lysine 36, and lysine 79 can activate transcription, whereas methylation of lysine 9 and lysine 27 is associated with the repression of transcription (Islam et al., 2011; Sanli et al., 2011). In *D. melanogaster*, the histone H3 lysine 27 trimethylation demethylase dUTX is essential for hormone-mediated transcription. It interacts with the ecdysone receptor, a nuclear hormone receptor complex, and regulates apoptosis and autophagy genes during ecdysone-driven PCD in salivary glands (Denton et al., 2013). This offers insights into the molecular mechanisms underlying autophagy modulation by histone methylation.

## Conclusion

Protein modifications play crucial roles in fundamental cellular physiological processes, including cell survival, autophagy, apoptosis, gene transcription, DNA repair, stress response, and genome stability (Lu and Gao, 2017; Han et al., 2018; Heo, 2019; Singh and Ostwal, 2019; Wang and Wang, 2019). Autophagy, a self-digestive and dynamic mechanism, is responsible for degrading long-lived proteins and malfunctioning organelles via lysosomal pathways (Klionsky et al., 2021). Furthermore, the autophagic process, which is essential for maintaining cellular homeostasis, plays a vital role in ensuring cell survival. Dysregulated autophagy patterns have been documented in various pathological conditions in eukaryotes, such as muscular disorders, cancer, tumorigenesis, aging, and neurodegeneration (Mizushima and Levine, 2010; Levine et al., 2011). Increasing evidence underscores the role of protein modifications throughout the autophagic process, from initiation to nucleation, elongation, completion, fusion, and even in the regulation of degradation and efflux of autophagic substrates (Wang and Wang, 2019). In this review, we emphasized the significance of PTMs such as acetylation/deacetylation, phosphorylation/dephosphorylation, ubiquitination/deubiquitination, lipidation, and methylation/demethylation in the regulation of autophagy in both *B. mori* and *D. melanogaster*. Several questions about insect autophagy remain unanswered and warrant further investigation: 1. Is there an intersection between PTMs that regulate autophagy in insects? 2. Do other PTMs, such as palmitoylation, lysine crotonylation, succinyllysine, and 2-hydroxyisobutyryl lysine, exert an influence on autophagy in insects? 3. Can high-resolution mass spectrometry and protein chips be used to measure and monitor PTMs in autophagy? 4. What are the effects of PTMs on structural modifications of proteins that regulate autophagy? 5. Why do PTMs that influence autophagy vary among different species? 6. What are the essential enzymes responsible for specific PTMs in insects? 7. Is it essential to discover new inhibitors/activators to target autophagy-related PTMs, with the aim of either restoring or inhibiting autophagy? 8. While Beclin 1/Atg5 is known to regulate autophagy associated with learning and memory in rats (Xu et al., 2016; Wang et al., 2021a), how do Atgs influence learning and memory in insects? A comprehensive understanding of PTMs in the autophagy regulation is imperative. Exploring the roles and mechanisms of PTMs not only provides profound insights into the regulation of autophagy processes but also underscores the significance of understanding the upstream pathways that influence PTMs. This knowledge will be pivotal in the development of pharmaceutical interventions targeting autophagy.
